# Comment to: Intensive support recommendations for critically-ill patients with suspected or confirmed COVID-19 infection

**DOI:** 10.31744/einstein_journal/2020CE5931

**Published:** 2020-07-31

**Authors:** Thiago Domingos Corrêa, Gustavo Faissol Janot de Matos, Bruno de Arruda Bravim, Ricardo Luiz Cordioli, Alejandra del Pilar Gallardo Garrido, Murillo Santucci Cesar de Assuncao, Carmen Silvia Valente Barbas, Karina Tavares Timenetsky, Roseny dos Reis Rodrigues, Hélio Penna Guimarães, Roberto Rabello, Frederico Polito Lomar, Farah Christina de La Cruz Scarin, Carla Luciana Batista, Adriano José Pereira, João Carlos de Campos Guerra, Bárbara Vieira Carneiro, Ricardo Kenji Nawa, Rodrigo Martins Brandão, Antônio Eduardo Pereira Pesaro, Moacyr Silva, Fabricio Rodrigues Torres de Carvalho, Cilene Saghabi de Medeiros Silva, Ana Claudia Ferraz de Almeida, Marcelo Franken, Marcele Liliane Pesavento, Raquel Afonso Caserta Eid, Leonardo José Rolim Ferraz

**Affiliations:** 1 Hospital Israelita Albert Einstein São PauloSP Brazil Hospital Israelita Albert Einstein, São Paulo, SP, Brazil.

Dear Editor,

In this issue of einstein journal (São Paulo), Corrêa et al., reported intensive support recommendations for critically-ill patients with suspected or confirmed infection by the new coronavirus (COVID-19).^(1)^ Based on preliminary demonstration of efficacy of chloroquine, the authors suggested the use of hydroxychloroquine in monotherapy or combined with a macrolide (azithromycin or clarithromycin), to treat severe patients with COVID-19, admitted to the intensive care unit (ICU), to inhibit *in vitro* replication of severe acute respiratory syndrome coronavirus 2 (SARS-CoV-2).^(2)^

However, evidence from observational studies^(3,4)^ involving hospitalized COVID-19 patients demonstrated the use of hydroxychloroquine in monotherapy^(3)^ or combined with a macrolide^(4)^was not associated with reduced mortality when compared to patients who did not receive such medications (controls). Yet, the use of hydroxychloroquine in monotherapy or combined with a macrolide may be associated with greater incidence of major cardiovascular complications.^(4)^

Therefore, based on new evidence available, we exclude the recommendation to use hydroxychloroquine in monotherapy or combined with a macrolide for treating inpatients with severe acute respiratory syndrome caused by COVID-19.
