# Supplementary material for: Comment to: Intensive support recommendations for critically-ill patients with suspected or confirmed COVID-19 infection
Source: Einstein (Sao Paulo). 2020 Jul 31;18:eCE5931. doi: 10.31744/einstein_journal/2020CE5931 (PMC7394010; doi:10.31744/einstein_journal/2020CE5931)
Supplement: Translation

# Translation: Comentário para: Recomendações de suporte intensivo para pacientes graves com infecção suspeita ou confirmada pela COVID-19

**Authors:** Thiago Domingos Corrêa, Gustavo Faissol Janot de Matos, Bruno de Arruda Bravim, Ricardo Luiz Cordioli, Alejandra del Pilar Gallardo Garrido, Murillo Santucci Cesar de Assuncao, Carmen Silvia Valente Barbas, Karina Tavares Timenetsky, Roseny dos Reis Rodrigues, Hélio Penna Guimarães, Roberto Rabello, Frederico Polito Lomar, Farah Christina de La Cruz Scarin, Carla Luciana Batista, Adriano José Pereira, João Carlos de Campos Guerra, Bárbara Vieira Carneiro, Ricardo Kenji Nawa, Rodrigo Martins Brandão, Antônio Eduardo Pereira Pesaro, Moacyr Silva, Fabricio Rodrigues Torres de Carvalho, Cilene Saghabi de Medeiros Silva, Ana Claudia Ferraz de Almeida, Marcelo Franken, Marcele Liliane Pesavento, Raquel Afonso Caserta Eid, Leonardo José Rolim Ferraz

**Affiliations:** 1 Brasil Hospital Israelita Albert Einstein, São Paulo, SP, Brasil.

Caro Editor,

Nesta edição da revista einstein (São Paulo), Corrêa et al., reportam recomendações de suporte intensivo para pacientes graves com infecção suspeita ou confirmada por doença pelo coronavírus 19 (COVID-19).^(1)^ Com base na demonstração preliminar da eficácia da cloroquina, os autores sugeriram, para tratamento de pacientes graves admitidos em unidade de terapia intensiva (UTI) com COVID-19,^(1)^ a utilização da hidroxicloroquina em monoterapia ou em associação com um macrolídeo (azitromicina ou claritromicina), de modo a inibir a replicação *in vitro* do coronavírus da síndrome respiratória aguda grave 2 (SARS-CoV-2).^(2)^

Entretanto, evidências provenientes de estudos observacionais^(3,4)^ envolvendo pacientes hospitalizados por COVID-19 demonstraram que a utilização de hidroxicloroquina em monoterapia^(3)^ ou em associação a macrolídeo^(4)^ não foi associada à redução da mortalidade quando comparada a pacientes que não receberam essas medicações (controles). Todavia, a utilização de hidroxicloroquina em monoterapia ou em associação a macrolídeo pode estar associada a maior incidência de complicações cardiovasculares maiores.^(4)^

Dessa forma, com base nas novas evidências disponíveis, nós retiramos a recomendação para utilização de hidroxicloroquina em monoterapia ou em associação a macrolídeo para tratamento de pacientes hospitalizados com síndrome respiratória aguda grave por COVID-19.
